# Expression and subcellular localisation of AID and APOBEC3 in adenoid and palatine tonsils

**DOI:** 10.1038/s41598-017-18732-w

**Published:** 2018-01-17

**Authors:** Noriko Seishima, Satoru Kondo, Kousho Wakae, Naohiro Wakisaka, Eiji Kobayashi, Makoto Kano, Makiko Moriyama-Kita, Yosuke Nakanishi, Kazuhira Endo, Tomoko Imoto, Kazuya Ishikawa, Hisashi Sugimoto, Miyako Hatano, Takayoshi Ueno, Miki Koura, Koichi Kitamura, Masamichi Muramatsu, Tomokazu Yoshizaki

**Affiliations:** 1 Division of Otolaryngology-Head and Neck Surgery, Graduate School of Medical Science, Kanazawa University, Kanazawa, Ishikawa Japan; 2 Department of Molecular Genetics, Graduate School of Medical Science, Kanazawa University, Kanazawa, Ishikawa Japan

## Abstract

Activation-induced cytidine deaminase (AID) and apolipoprotein B mRNA-editing catalytic polypeptide 3 (A3) family are cytidine deaminases that play critical roles in B-cell maturation, antiviral immunity and carcinogenesis. Adenoids and palatine tonsils are secondary lymphoid immune organs, in which AID and A3s are thought to have several physiological or pathological roles. However, the expression of AID or A3s in these organs has not been investigated. Therefore, we investigated the expression profiles of AID and A3s, using 67 samples of adenoids and palatine tonsils from patients, with reverse transcription quantitative polymerase chain reaction (RT-qPCR) and immunohistochemical analyses. *AID* and *A3s* expression levels in the adenoids and the palatine tonsils of the same individual significantly correlated with each other. Of note, *AID* expression level in the adenoids negatively correlated with the age (*r* = −0.373, *P* = 0.003). The younger group with adenoid vegetation and tonsillar hypertrophy showed more abundant AID expression than the older group with recurrent tonsillitis and peritonsillar abscesses (*P* = 0.026). Moreover, immunohistochemical analysis revealed the distribution of AID and A3s in the epithelial cells as well as germinal centres. The localisation of AID expression and its relation to age may contribute to adenoid vegetation and inflammation.

## Introduction

Apolipoprotein B mRNA-editing catalytic polypeptide (APOBEC) enzymes are a family of cytidine deaminases that convert cytosine in DNA and/or RNA into uracil. In humans, the APOBEC family is composed of at least 11 members, including activation-induced cytidine deaminase (AID) and APOBEC1, 2, 3 A, 3B, 3 C, 3D, 3 F, 3 G, 3 H and 4^1,2^. These proteins, particularly AID and APOBEC3s (A3s), are important for both innate and adaptive immune responses. AID is expressed in B cells and is essential for antibody diversification, including somatic hypermutation and class switch recombinations that occur in germinal centres (GCs)^3,4^. Furthermore, Epstein-Barr virus infected B-cells induces expression of AID^5^. AID is also found in non-B cells and is induced by the stimulation of inflammatory cytokines that are involved in some viral infections such as hepatitis C virus and hepatitis B virus in hepatocytes^6–10^. Further, aberrant expression of AID leads to the accumulation of genetic changes and carcinogenesis, including oral squamous cell carcinoma, B-cell lymphoma, gastric cancer and skin cancer^11–15^. Similarly, A3s act as antiretroviral factors, by introducing frequent C-to-U conversion in newly generated viral DNA^16,17^. A3 enzymes localise to the cytoplasm and/or nucleus, enabling the protection of both compartments by the restriction of nuclear or cytoplasmic replicating elements. Eventually they show antiviral effects on DNA viruses such as hepatitis B^18–21^.

Adenoids and palatine tonsils are lymphoid tissues located in the pharynx that play an important role in host defence against pathogens invading the upper respiratory tract^22^. In GCs, B cells differentiate into plasma and memory B cells that secrete high-affinity antibodies and endow individuals with immunological memory^23^. Almost all persons have adenoid vegetation and hypertrophic palatine tonsils during childhood. The size of the adenoids and palatine tonsils decreases gradually and spontaneously with age; hence, these tissues are rudimentary in most adults^24^. Adenoid vegetation or hypertrophic palatine tonsils often cause clinical symptoms such as nasal obstruction, snoring and obstructive sleep apnoea. Complications of adenoid vegetation, hypertrophic palatine tonsils, or recurrent tonsillitis frequently force patients to undergo either tonsillectomy or adenotonsillectomy. However, the pathophysiology of these diseases remains largely unknown.

To elucidate the pathogenic role of AID or A3s expression on adenoid vegetation, tonsillar hypertrophy, or tonsillitis, we examined their expression profile and distribution in surgically treated adenotonsillar organs with various pathological backgrounds.

## Results

### Patient characteristics

The characteristics of all patients for RT-qPCR analysis are shown in Table 1. The mean age of patients was 18 years (range, 2–89 years). All 14 patients who underwent adenotonsillectomy were <16 years old and suffered obstructive sleep apnoea because of adenoid vegetation, although 29 patients (91%) <16 years old, with either adenoid vegetation or tonsillar hypertrophy, underwent adenoidectomy or tonsillectomy. The 35 patients ≥16 years old underwent tonsillectomy. Among them, 18 patients (51%) received tonsillectomy because of recurrent tonsillitis, 3 patients (9%) for repeated peritonsillar abscess and 9 patients (26%) for focal tonsils.

**Table 1:** Patient characteristics.

	Total		≥16 years		<16 years	
	*n*	Mean ± SD	*n*	Mean ± SD (range)	*n*	Mean ± SD (range)
Age, years	67	18 ± 17.3	35	27 ± 15.3 (16–89)	32	5.5 ± 2.53 (2–13)
Gender						
Female	26		14		12	
Male	41		21		20	
Types of operation performed						
Adenotonsillectomy	14		0		14	
Tonsillectomy	53		35		18	
Disease						
Adenoid vegetation and tonsillar hypertrophy	22		0		22	
Tonsillar hypertrophy	10		3		7	
Recurrent tonsillitis	20		18		2	
Repeated peritonsillar abscess	3		3		0	
IgA nephropathy	7		7		0	
Palmoplantar pustulosis	2		2		0	
Others	3		2		1	

*n*, number of patients; SD, standard deviation.

We performed immunohistochemical analysis on 15 adenoid specimens and 14 palatine tonsil specimens from the same 15 individuals. The mean age of the 15 patients was 32 years (range, 5–19 years), including nine patients aged ≥16 years (range, 26–89 years) and six patients aged <16 years (range, 5–13 years).

### Expression level of AID/A3s in the adenoid and palatine tonsils of each individual

**Figure 1:**
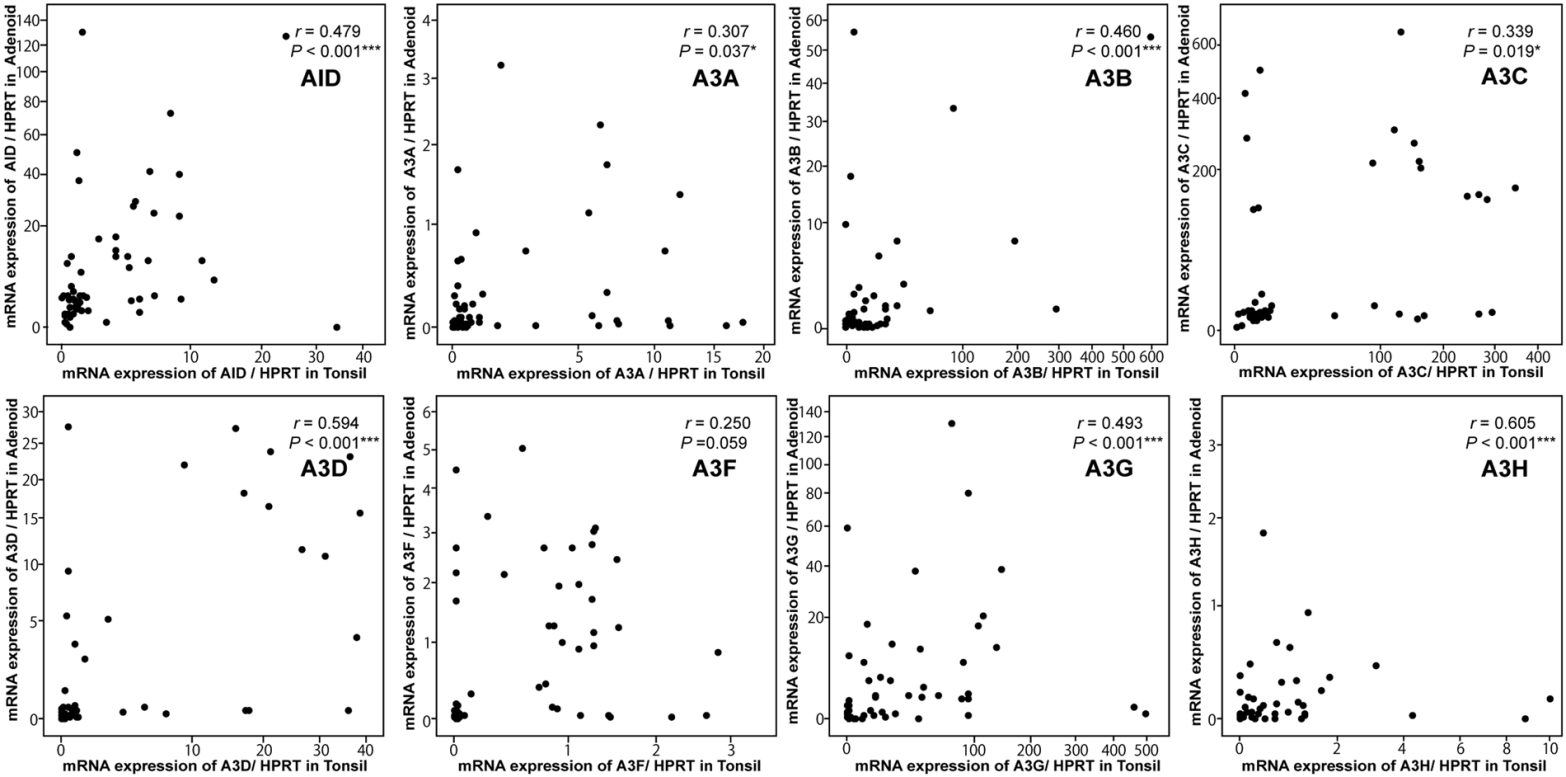
Correlation between AID and A3s expression levels in the adenoids and palatine tonsils. *P*: P-value; *r*: correlation coefficient; *P < 0.05; **P < 0.01; ***P < 0.001, as determined by Spearman rank correlation coefficient.

**Figure 2:**
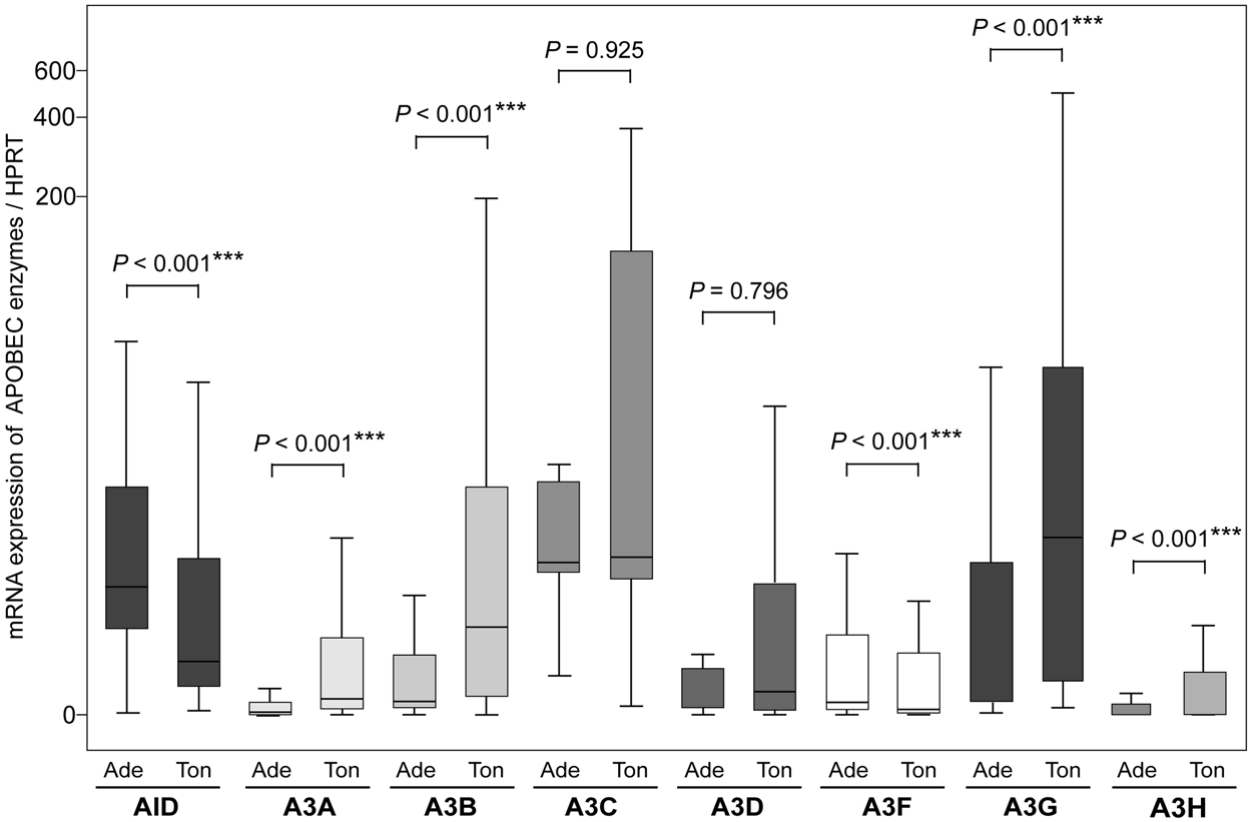
Comparison of AID and A3s expression levels in the adenoids and palatine tonsils. Comparison of the median values for AID or A3s expression between adenoids and tonsils. *P*, P-value; ***P < 0.001, as determined by Mann–Whitney *U* test.

Initially, we determined the expression level of *AID/A3s* in the adenoids and palatine tonsils of each individual using RT-qPCR. We compared the expression level of *AID/A3* mRNA in the adenoids with that of the palatine tonsils in each individual (Fig. 1). The level of all *AID/A3s* in the adenoids, except for *A3F*, significantly correlated with those in the palatine tonsils. The levels of *AID* and *A3F* were higher in the adenoids than that in the palatine tonsils, whereas the levels of *A3A, A3B, A3G* and *A3H* were higher in the palatine tonsils (Fig. 2).

We further examined the correlation between the levels of *AID/A3* mRNA in the adenoids and palatine tonsils. In the adenoids, there was no correlation between the levels of *AID* and any of the *A3s* (Table 2), whereas the levels of *A3s* were largely correlated with each other (Table 2). By contrast, in the palatine tonsils, *AID* levels significantly correlated with *A3s* (Table 3). In the adenoids, *A3s* levels moderately correlated with each other.

**Table 2:** Comparison of each AID and A3s expression in the adenoids and palatine tonsils.

		AID	A3A	A3B	A3C	A3DE	A3F	A3G
**(A) In the adenoids**								
A3A	*r P*	0.023 0.860						
A3B	*r P*	0.182 0.167	0.500 <0.001***					
A3C	*r P*	0.236 0.071	0.424 <0.001***	0.623 <0.001***				
A3D	*r P*	0.172 0.192	0.072 0.589	0.347 0.008**	0.690 <0.001***			
A3F	*r P*	0.204 0.121	0.654 <0.001***	0.461 <0.001***	0.689 <0.001***	0.362 0.004**		
A3G	*r P*	0.220 0.094	0.664 <0.001***	0.501 <0.001***	0.501 <0.001***	0.179 0.176	0.685 <0.001***	
A3H	*r P*	0.170 0.198	0.688 <0.001***	0.370 0.004**	0.480 <0.001***	0.089 0.503	0.702 <0.001***	0.888 <0.001***
**(B) In the palatine tonsils**								
A3A	*r P*	0.364 0.003**						
A3B	*r P*	0.302 0.015*	0.742 <0.001***					
A3C	*r P*	0.679 <0.001***	0.573 <0.001***	0.423 <0.001***				
A3D	*r P*	0.468 <0.001***	0.759 <0.001***	0.391 <0.001***	0.596 <0.001***			
A3F	*r P*	0.744 <0.001***	0.491 <0.001***	0.295 0.018*	0.603 <0.001***	0.423 <0.001***		
A3G	*r P*	0.410 <0.001***	0.535 <0.001***	0.649 <0.001***	0.380 0.002**	0.225 0.077	0.557 <0.001***	
A3H	*r P*	0.509 <0.001***	0.640 <0.001***	0.548 <0.001***	0.495 <0.001***	0.383 0.002**	0.727 <0.001***	0.869 <0.001***

*n*, patient number; *P*, *P*-value; *r*, correlation coefficient; *P < 0.05; **P < 0.01; ***P < 0.001.

**Table 3:** Association of subcellular location of AID and A3s with region of adenoids and palatine tonsils. The subcellular location of immunoreactive cells was separately evaluated for nucleus and cytoplasm.

		Adenoid (*n* = 14)		Palatine tonsil (*n* = 15)			
		Epithelium (mean ± SD)	*P*	Epithelium (mean ± SD)	*P*	Folliclular GC (mean ± SD)	*P*
AID	*N+C* score	3.37 ± 0.89		3.26 ± 0.33		2.54 ± 1.19	
	*N* score*C* score	1.44 ± 0.75	0.029*	1.50 ± 0.83	0.511	0.85 ± 0.62	0.001**
		1.93 ± 0.25		1.76 ± 0.56		1.69 ± 0.72	
A3A	*N+C* score	2.17 ± 0.35		1.97 ± 0.93		1.21 ± 1.51	
	*N* score*C* score	1.88 ± 0.20	<0.001***	1.59 ± 0.57	<0.001***	0.78 ± 0.76	0.094
		0.28 ± 0.35		0.38 ± 0.56		0.42 ± 0.80	
A3B	*N+C* score	2.41 ± 1.27		2.58 ± 1.23		1.79 ± 1.24	
	*N* score*C* score	0.75 ± 0.81	0.038*	0.88 ± 0.76	0.040*	1.36 ± 0.55	0.008*
		1.67 ± 0.62		1.70 ± 0.60		0.51 ± 0.72	
A3C	*N+C* score	2.86 ± 1.06		2.00 ± 0.92		1.95 ± 1.14	
	*N* score*C* score	1.04 ± 0.77	0.006**	0.35 ± 0.60	<0.001***	0.45 ± 0.44	0.002**
		1.82 ± 0.41		1.64 ± 0.51		1.50 ± 0.80	
A3D	*N+C* score	0.62 ± 0.81		0.35 ± 0.56		0.92 ± 0.74	
	*N* score*C* score	0	0.061	0	0.056	0.02 ± 0.08	<0.001***
		0.62 ± 0.81		0.35 ± 0.56		0.90 ± 0.72	
A3F	*N+C* score	2.05 ± 1.08		2.42 ± 0.79		1.83 ± 1.07	
	*N* score*C* score	0.33 ± 0.61	0.011*	0.79 ± 0.56	0.003**	0.29 ± 0.70	0.007*
		1.71 ± 0.76		1.75 ± 0.39		1.54 ± 0.59	
A3G	*N+C* score	2.37 ± 1.11		1.73 ± 0.84		2.33 ± 0.59	
	*N* score*C* score	0.62 ± 0.85	0.001**	0.23 ± 0.40	<0.001***	0.52 ± 0.38	<0.001***
		1.75 ± 0.44		1.50 ± 0.61		1.81 ± 0.31	
A3H	*N+C* score	3.11 ± 0.94		2.78 ± 0.97		3.31 ± 0.80	
	*N* score*C* score	1.28 ± 0.64	0.010*	1.00 ± 0.67	0.002**	1.30 ± 0.80	0.024**
		1.82 ± 0.37		1.78 ± 0.36		2.00	

Expression score 0, <10% immunoreactive cells; expression score 1, 10%–50% immunoreactive cells; expression score 2, >50% immunoreactive cells.

*N* + *C* score, the total expression score; *N* score, the nucleus expression score; *C* score, the cytoplasm expression score.

*n*, patient number; SD, standard deviation.

*P*, *P*-value for correlation of *N* score and *C* score by the Mann–Whitney *U* test.

*P < 0.05; **P < 0.01; ***P < 0.001.

These results indicate that the expression level of *AID/A3s* in the adenoids correlated with that in the palatine tonsils for each individual, and that the adenoids and the palatine tonsils have distinct expression profiles for *AID/A3s*.

### Association between age and expression level of AID/A3s in the adenoids and palatine tonsils

**Figure 3:**
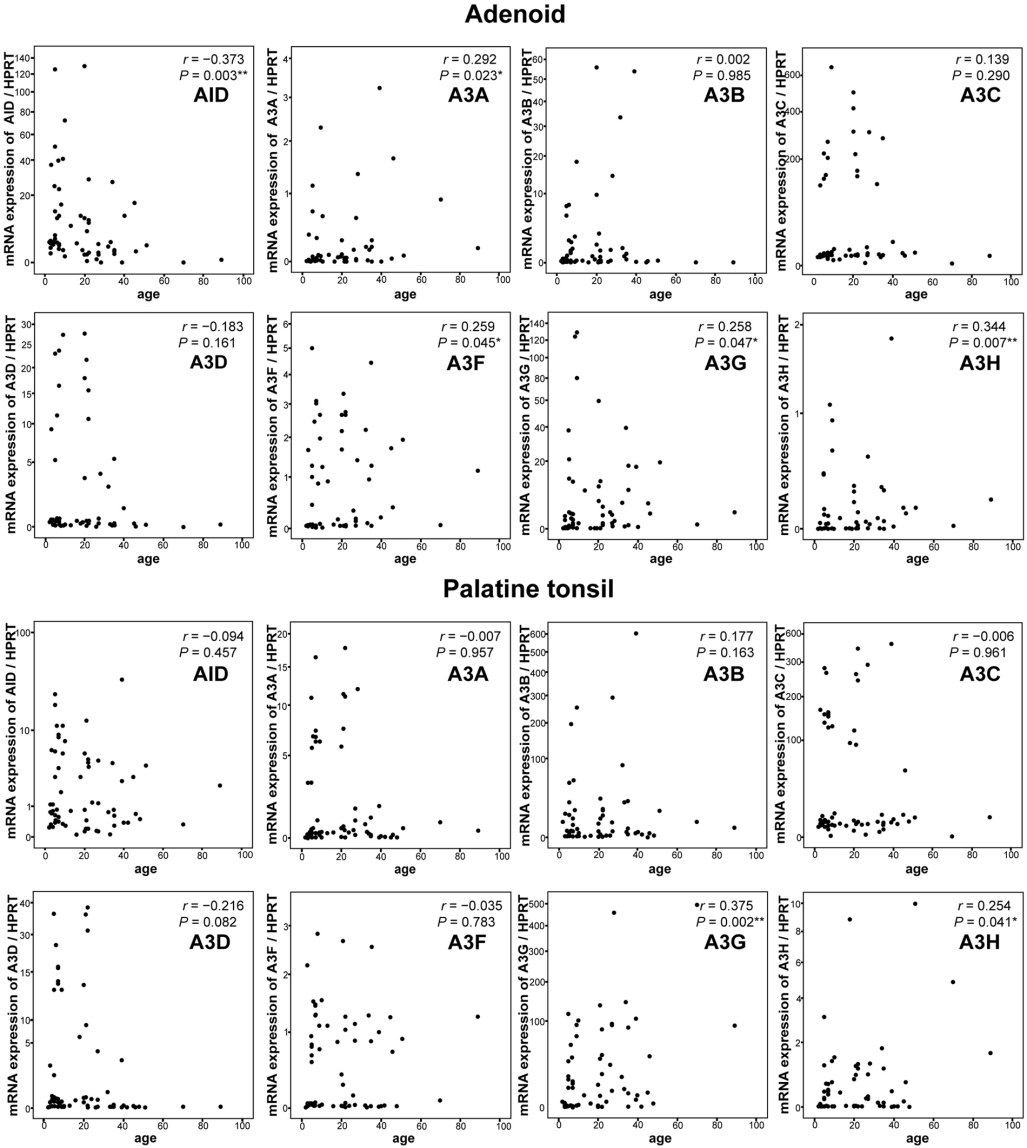
Correlation between AID and A3s expression levels and age in the adenoids and palatine tonsils. *P*, *P*-value; *r*, correlation coefficient; *P < 0.05; **P < 0.01, as determined by Spearman rank correlation coefficient.

Next, we examined the association between *AID/A3s* expression levels and age. In the adenoids, even though the regression coefficient suggests a relatively weak relationship, *AID* negatively correlated with age, whereas *A3A, A3F, A3G* and *A3H* had significant and positive correlations with age (Fig. 3). In the palatine tonsils, *A3G* and *A3H*, but not *AID*, significantly correlated with the age at operation (Fig. 3). This indicated that *A3s* expression levels remained stable or increased with age, whereas *AID* expression levels in the adenoids decreased with age.

**Figure 4:**
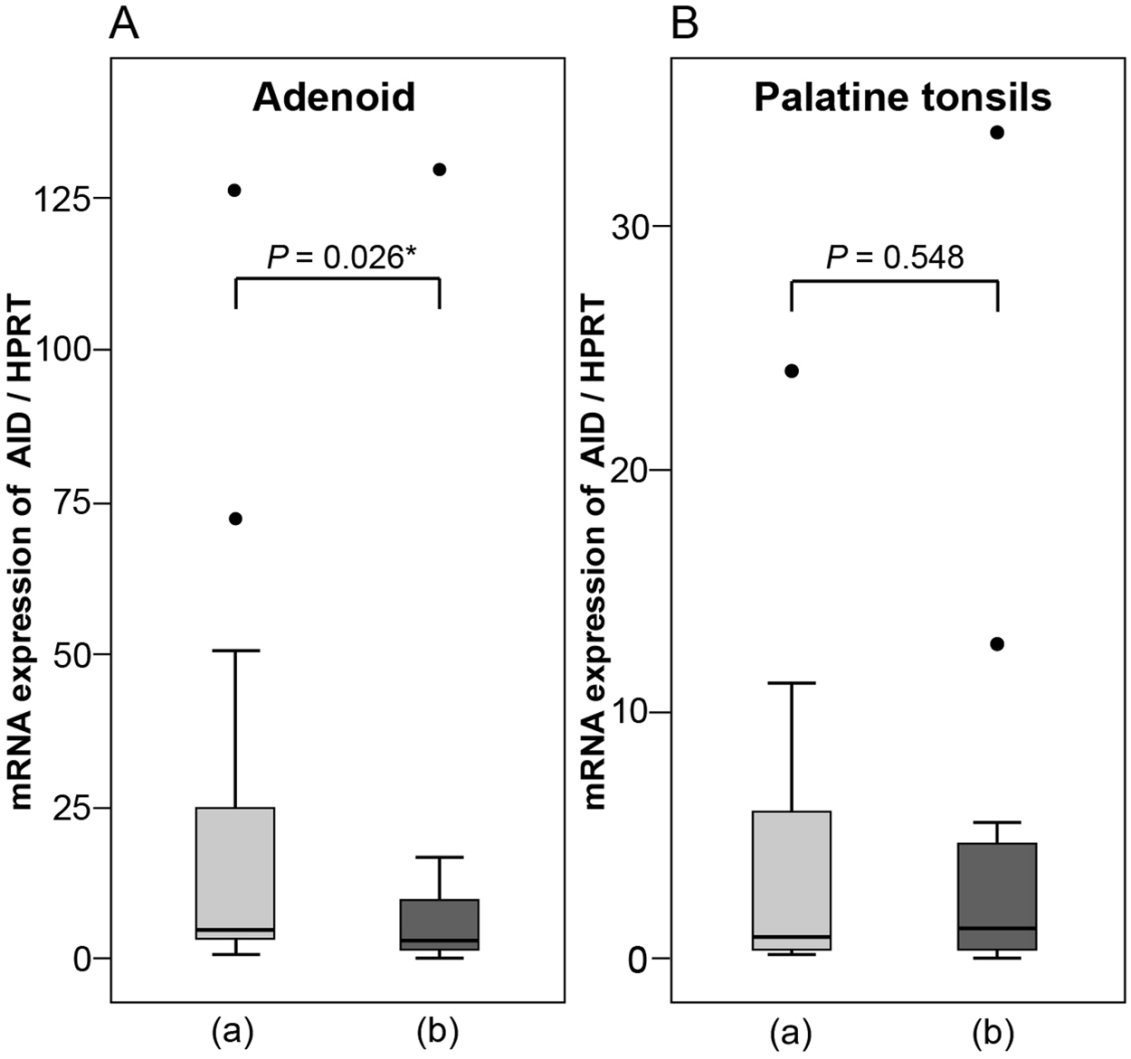
Comparison of AID expression levels in the adenoids and palatine tonsils of two groups. (**A**) Adenoid vegetation and tonsillar hypertrophy; <16 years old, n = 29 and (**B**) Recurrent tonsillitis and repeated peritonsillar abscess; ≥16 years old, n = 21. *n*, number of patients; *P*, *P*-value; *P < 0.05, as determined by Mann–Whitney *U* test.

Moreover, we investigated associations between *AID/A3s* expression levels and pathophysiology of the adenoids and the palatine tonsils. Figure 4 shows a comparison of *AID* expression levels between two groups in these tissues. In the adenoids, *AID* expression level of adenoid vegetation and tonsillar hypertrophy in patients <16 years old (a) was higher than that observed in patients with recurrent tonsillitis and peritonsillar abscesses who were ≥16 years old (b) (Fig. 4A). In the palatine tonsils, there was no statistically significant difference in *AID* expression between the two groups (Fig. 4B). Moreover, there was no statistically significant difference in the expression of any *A3s* between the two groups in the adenoids and the palatine tonsils (see Supplementary Fig. S1). This suggested that the age-related expression of *AID*, and reason for the operation, affected the volume of tissues in the adenoids but not in the palatine tonsils.

### Subcellular localisation of AID/A3s in the adenoids and palatine tonsils

We performed immunohistochemical analysis to assess the subcellular localisation of AID/A3s in the adenoids and the palatine tonsils.

**Figure 5:**
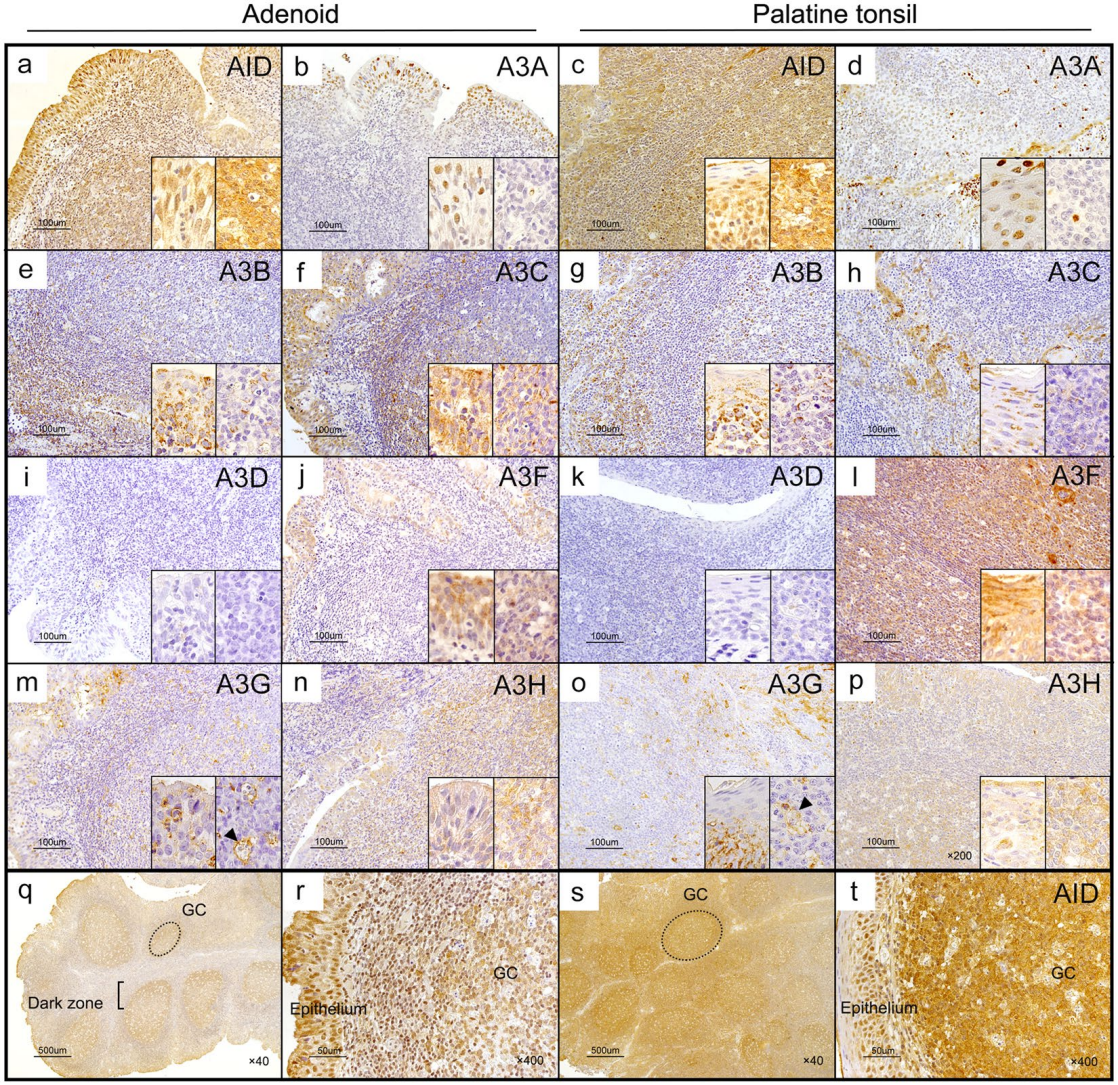
Subcellular localisation of AID and A3s in adenoids and palatine tonsils. Representative images of immunohistochemical analyses of AID/A3s. An adenoid (**a,b,d,f,i,j,m,n,q** and **r**) and a palatine tonsil (**c,d,g,h,k,l,o,p,s** and **t**) from a 5-year-old boy, who suffered from sleep apnoea and underwent adenotonsillectomy, were analysed. The specimens were stained using antibodies against AID (**a**,**c** and **q**–**t**), A3A (**b** and **d**), A3B (**e** and **g**), A3C (**f** and **h**), A3D (**i** and **k**), A3F (**j** and **l**), A3G (**m** and **o**) and A3H (**n** and **p**). The inset figures indicate epithelium (left) and GC (right). Original magnifications: 200 × (**a**–**p**), 40 × (q and s), 400 × (**r** and **t**) and 600 × (inset figures). Scale bar: 100 μm (**a**–**p**), 500 μm (**q** and **s**) and 50 μm (r and t).

AID expression was detected in the dark zone of GCs in both the adenoids and palatine tonsils, as reported previously^25–29^ (Fig. 5a,c,q and s). Unexpectedly, AID expression in the epithelial cells was comparable with that in the GCs (Fig. 5r and t). The mean total expression scores were 3.37 for the epithelium of the adenoids, 3.26 for the epithelium, and 2.54 for the follicular GC in the tonsils (Table 3).

For A3s, the epithelial cells express A3A, A3B, A3C, A3F, A3G and A3H, whereas the GCs express A3B, A3C, A3F, A3G and A3H (Table 3 and Fig. 5b and d–p). Of note, A3G was found in tangible body macrophages (Fig. 5m and o). Crypts expressed A3A, A3B, A3C, A3F, A3G and A3H, while infiltrated haematopoietic cells expressed A3A. A3D was poorly detected in the epithelium, GCs, or crypts but was detected in vessels.

The subcellular localisation of AID/A3s is summarised in Table 3. All were expressed in both the nucleus and cytoplasm although AID, A3B, A3C, A3D, A3G, A3F and A3H were abundant in the cytoplasm and A3A was rich in the nucleus (Table 3 and Fig. 5). A3B was expressed abundantly in the cytoplasm of the epithelium and in the nucleus of the GC.

## Discussion

In this study, we examined the expression and distribution of APOBEC enzymes, particularly AID and A3s, in the adenotonsillar tissues. *AID/A3s* expression were weakly to moderately correlated with each other within a single subject (Fig. 1). Their expression profile differed between the adenoids and palatine tonsils (Fig. 2). *AID* was expressed independently from *A3s* in the adenoids; however, *AID/A3s* were correlated with each other in the adenoids and the palatine tonsils (Table 2).

Furthermore, there was a negative correlation between age and *AID/A3* expression in the adenoids and a positive correlation between age and *AID/A3s* in the palatine tonsils, respectively (Fig. 3). There was no statistically significant difference between age and *AID* in the palatine tonsils or *A3s* in the adenoids and palatine tonsils, but there was statistically significant difference between age and *AID* in the adenoids (Fig. 3). Immunohistochemical analysis revealed abundant AID expression in the epithelium, comparable with that observed in the GCs (Fig. 5). These results revealed a comprehensive profile of AID/A3s expression in adenotonsillar tissues. However, the samples analysed in this study were all obtained from the patients. The etiologies differed between the younger and the older patients who underwent surgery. Generally, the expression of AID and A3s is affected by the transcriptional factors such as NF-κB that acts downstream of inflammatory cytokines such as TNFα^7–10^. Thus, the degree of inflammation and infection, as well as the age of the patients at operation, might have affected the results obtained in this study.

AID is predominantly cytoplasmic and shuttles between the nucleus and cytoplasm^30^, whereas A3A, A3C and A3H are found in both the nucleus and cytoplasm^16,18,24^; this was not consistent with our results. In contrast, previous reports indicate that A3B localises in the nucleus, whereas A3D, A3F and A3G are found in the cytoplasm^16,18,24^, which is not consistent with our data that showed that A3s localised in both the nucleus and cytoplasm (Table 3 and Fig. 5). This discrepancy could be attributed to the different cellular contexts among murine fibroblasts, adenotonsillar epithelial or immune cells, HeLa and 293 T cells used in their studies. Further studies are necessary to identify undetermined factors that regulate the subcellular localisation of A3s in adenotonsillar tissues.

Mattila *et al*.^31^ reported that the proportion of B cells producing immunoglobulin decreases with ageing in adenotonsillar tissues, particularly in adenoids. Age-related decreases coincide with decreased B-cell population or production of immunoglobulin. Because AID is a master gene for B-cell maturation^3,4^, the inverse correlation between *AID* expression in adenoid vegetation and ageing (Figs 3 and 4A) may reflect decreased maturation of B cells with ageing.

Onal *et al*.^32^ reported that the apoptosis of lymphocytes in the GC of tonsillar hypertrophy increased compared with chronic tonsillitis. *AID* expression in tonsillar hypertrophy at a younger age was not higher than that in repeated tonsillitis (Fig. 4B), which may reflect the increased apoptosis of B cells in GC. Further studies are necessary to clarify the role of AID in adenoid vegetation and tonsillar hypertrophy.

Aberrant AID or A3s expression is associated with inflammation. AID is ectopically induced in gastric epithelium by *Helicobacter pylori* infection via NF-κB^10^ or inflammatory cytokines, such as TNFα or IL-1β^9,10,33^. Further, A3s are induced by interferons and lipopolysaccharides^8,21,34^. We found abundant AID and A3s expression in the epithelial cells of the adenoids and the palatine tonsils, compared with that in GCs (Fig. 5a,c,r and t). *A3* expression positively correlated with age in the palatine tonsils (Table 3) although there was no statistically significant difference between the palatine tonsils with hypertrophy and those with repeated inflammation (Supplementary Fig. S1). Therefore, it is intriguing to speculate that repeated inflammation by repetitive infection, whether clinical or subclinical, induces aberrant AID or A3s expression.

In conclusion, we describe the unique distribution of AID and A3s and their association with age as well as surgical rationale for adenotonsillar tissues. Further investigation is required to reveal the physiological or pathological consequences of AID and A3s expression in adenotonsillar tissues.

## Methods

### Organs

Adenoid and palatine tonsil samples were collected from 67 patients who underwent adenotonsillectomy or tonsillectomy between October 2012 and March 2014 in the Division of Otolaryngology and Head and Neck Surgery at the Kanazawa University Hospital in Kanazawa and five other branch hospitals. The research protocol was approved by the ethics committees of the Kanazawa University Hospital and the five other participating hospitals. All experiments were carried out in accordance with the relevant guidelines and regulations. Informed consent was obtained from all patients and guardians prior to enrolment. Adenoids from patients who underwent tonsillectomy were collected by biopsy. None of the patients suffered any acute inflammation.

### RNA extraction and RT-qPCR

RNA was extracted from adenoid and palatine tonsil tissues using the RNeasy Plus Mini Kit (QIAGEN, Tokyo, Japan) according to the manufacturer’s instructions. Total RNA (5 μg) was reverse transcribed using the SuperScript III First-Strand Synthesis System for RT reactions (Thermo Fisher Scientific, MA, USA). cDNA was amplified using specific primers for A3A, A3B, A3C, A3D, A3F, A3G, A3H, AID and hypoxanthine-guanine phosphoribosyltransferase as described previously^7,21^.

### Statistical analysis for RT-qPCR

*AID/A3s* expression levels are shown as median values. *AID/A3s* expression levels between adenoids and palatine tonsils were compared using the Mann–Whitney *U* test and the Spearman rank correlation coefficient. The association between expression levels of *AID/A3s* and age was analysed using the Spearman rank correlation coefficient. Moreover, we divided patients into two groups and compared the expression levels of *AID/A3s* in the adenoids and palatine tonsils between both groups using the Mann–Whitney *U* test: (a) adenoid vegetation and tonsillar hypertrophy, age < 16 years, (b) recurrent tonsillitis and repeated peritonsillar abscess, age ≥ 16 years.

### Immunohistochemical analysis

The expression of AID/A3s was immunohistochemically examined in 15 adenoids and 14 palatine tonsils among 67 patients for RT-qPCR analysis. These 15 adenoids and 14 palatine tonsils were obtained from the same individuals. Consecutive 3-μm sections were cut from formalin-fixed, paraffin-embedded blocks of adenoids and palatine tonsils.

To detect AID, monoclonal antibody clone EK2 5G9 was used (1:5000, Cell Signaling Technology Inc., Boston, USA). Immunohistochemical staining was performed using the Vectastain Elite ABC kit (Vector Laboratories Inc., Burlingame, USA)^11^. To determine A3s expression, tissue sections were incubated with monoclonal antibodies against A3s. After washing with PBS, the sections were exposed to Envision^+^ secondary antibody (Dako, Glostrup, Denmark) and counterstained with hematoxylin. Antibodies for A3A, A3G and A3H are described previously^21,35^. Anti-A3C antibody clone 10591-1-AP (1:50, Proteintech, Chicago, USA), anti-A3B antibody clone ab184990 (1:100, Abcam, Cambridge, UK), anti-A3D antibody clone HPA055116 (1:200, Sigma Aldrich, St. Louis, USA), and anti-A3F antibody clone H00200316-A01 (1:50, abnova, Taipei, Taiwan) were used.

We examined cross-reactivity between anti-AID and A3 antibodies described above by Western blotting. (Supplementary Methods and Supplementary Table S1). The results showed no cross-reactivity between each anti-AID and A3 antibodies except for anti-A3A and anti-A3F antibodies. The anti-A3A antibody mainly reacted with A3A protein and reacts very faintly with A3D protein, which is oeverexpressed in 293FT cells. In contrast, the anti-A3F antibody mainly reacted A3F protein and reacts very faintly with overexpressed A3C protein (Supplementary Fig. S2).

### Evaluation of the specimens and statistical analysis

Stained sections were evaluated by two authors (NS and SK), who were independently blinded to the clinical data. The subcellular localisation of immunoreactive cells was separately evaluated for the nucleus and cytoplasm. The adenoid specimens were only evaluated with a part of epithelium because the specimens were tiny and only a few samples included follicles. The palatine tonsil specimens were separately evaluated with a part of the epithelium and GC. The average frequency of immunoreactive cells was calculated after counting the number of immunoreactive cells and the total number of epithelial cells or lymphocytes at three different high-powered fields (400×). The average percentage of immunoreactive cells in the nucleus and cytoplasm was defined as the *N* expression score and *C* expression score, respectively. Further, we calculated the total expression score. These three scores were used for statistical analysis. The average frequency of immunoreactive cells was classified into three groups to determine localisation of nucleus and cytoplasm: expression score 0, <10% immunoreactive cells; expression score 1, 10%–50% immunoreactive cells; expression score 2, >50% immunoreactive cells^36^. We evaluated the number of cells expressing AID/A3s to determine the expression level of these enzymes.

## Electronic supplementary material

Supplementary Information
