# Supplementary material for: Expression and subcellular localisation of AID and APOBEC3 in adenoid and palatine tonsils
Source: Sci Rep. 2018 Jan 17;8:918. doi: 10.1038/s41598-017-18732-w (PMC5772672; doi:10.1038/s41598-017-18732-w)
Supplement: Supplementary file 1 — Supplementary Information [file 41598_2017_18732_MOESM1_ESM.pdf]

Supplementary Information

Expression and subcellular localisation of AID and APOBEC3 in adenoid and palatine tonsils

Noriko Seishima[1], Satoru Kondo[1,]*, Kosho Wakae[2], Naohiro Wakisaka[1], Eiji Kobayashi[1], Makoto Kano[1], Makiko Moriyama-Kita[1], Yosuke Nakanishi[1], Kazuhira Endo[1], Tomoko Imoto[1], Kazuya Ishikawa[1], Hisashi Sugimoto[1], Miyako Hatano[1], Takayoshi Ueno[1], Miki Koura[2], Koichi Kitamura[2], Masamichi Muramatsu[2], and Tomokazu Yoshizaki[1]

Division of Otolaryngology-Head and Neck Surgery[1] and Department of Molecular Genetics[2], Graduate School of Medical Science, Kanazawa University, Kanazawa, Ishikawa, Japan

Corresponding author*: Satoru Kondo

Division of Otolaryngology-Head and Neck Surgery, Graduate School of Medical Science, Kanazawa University, 13-1 Takara-machi, Kanazawa 920-8640, Ishikawa, Japan

Phone: 81.76.265.2413; E-mail: ksatoru@med.kanazawa-u.ac.jp

Supplementary Figure S1: Comparison of A3 expression levels in the adenoids and palatine tonsils of the two groups.

(a) Adenoid vegetation and tonsillar hypertrophy; <16 years old.

(b) Recurrent tonsillitis and repeated peritonsillar abscess; ≥16 years old

n, number of patients; *P*, *P*-value, as determined by Mann–Whitney *U* test.

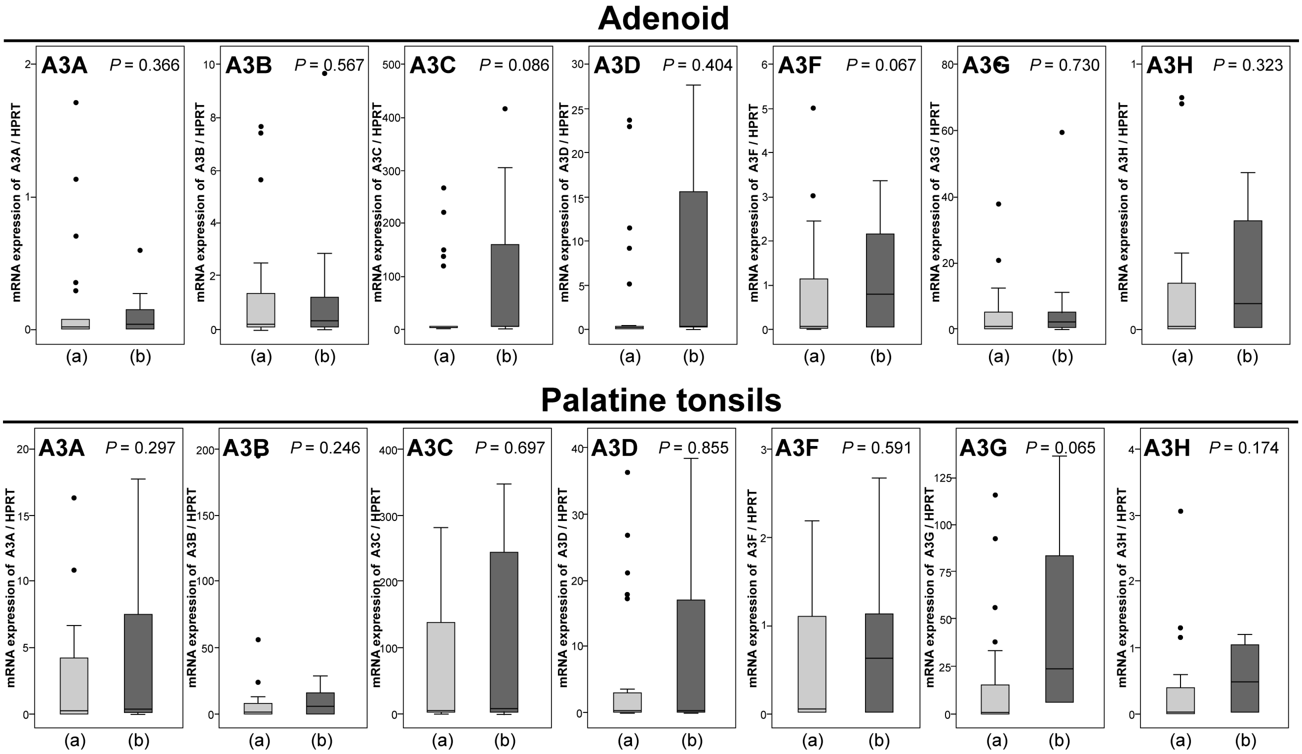

Supplementary Methods: Cell cultures, transfection and Western blotting

We transfected FLAG-tagged AID, A3A A3C, A3D, A3F, A3G, A3H, A1, A2 and GAPDH and HA-tagged A3B plasmid into 293T cells and then cultured as described previously[7,21]. The vectors used in this study are listed on Supplementary Table S1.

We performed Western blotting using a standard method as described previously[7,21]. Antibodies for AID, A3A, A3B, A3C, A3D, A3F, A3G and A3H used in this study are described in the main article. The rabbit anti-GAPDH (G9545, Sigma Aldrich, St. Louis, USA) and mouse anti-FLAG (M2, Sigma Aldrich, St. Louis, USA) and anti-HA (ab-hatag, InvivoGen, San Diego, USA) were also used.

Supplementary Table S1: Vector list

Name	Description
pFLAGhAID	Previously mentioned (7)
pFLAGhA3A	Previously mentioned (7) (37)
phA3B-HA	Cat number, 11090 from The NIH AIDS Research and Reference Reagent Program, Division of AIDS.
pFLAGhA3C	Previously mentioned (7) (37)
pFLAGhA3D	Human A3D ORF from pcDNA3.1-APOBEC3DE-V5-6xHIS (Cat number 11433 from The NIH AIDS Research and Reference Reagent Program, Division of AIDS) was inserted in a multiple cloning site of pCMV3Tag3B.
pFLAGhA3F	Previously mentioned (7) (37)
pFLAGhA3G	Previously mentioned (7) (21) (37)
pFLAGhA3H	Previously mentioned (37)
pFLAGhA1	Human APOBEC1 ORF (NM_001304566.1) was inserted in a multiple cloning site of pCMV3Tag1C.
pFLAGhA2	Human APOBEC2 ORF (NM_006789.3) was inserted in a multiple cloning site of pCMV3Tag3B.

Supplementary Figure S2: Western blotting of antibodies of AID and A3s

Expression of AID, A3s, A1 and A2 was examined using anti-AID, A3 antibodies used in the immunohistochemical analysis in this study. Western blotting of GAPDH was also conducted to estimate transfection efficiency and protein loading. The arrows point the minor cross-reactivity of A3D with anti-A3A antibody and A3C with anti-A3F antibody, respectively, α, anti-.

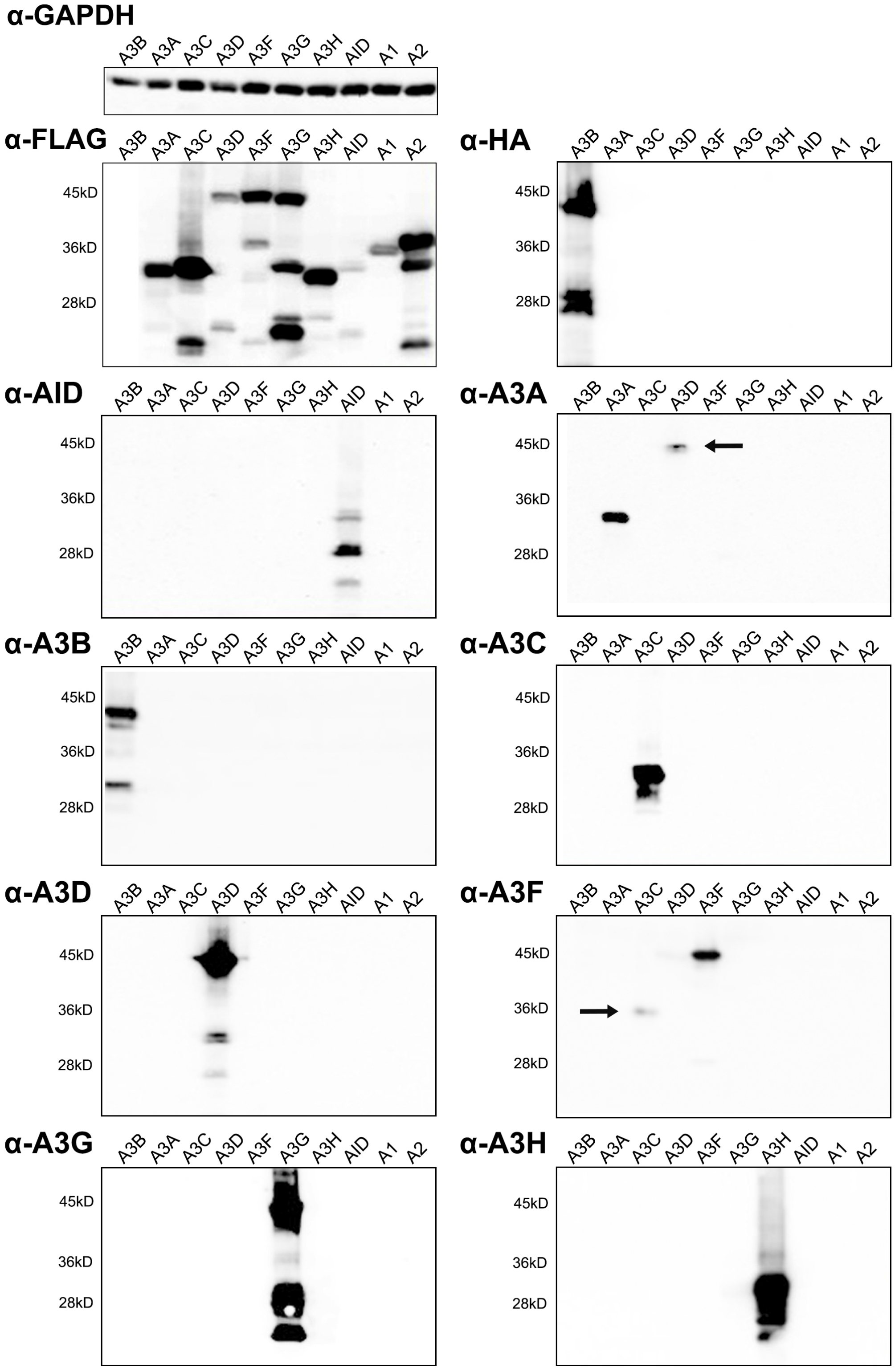

Reference

References 7 and 21 are same with those in the main article reference list.

7. Liang, G. *et al.* TGF-β suppression of HBV RNA through AID-dependent recruitment of an RNA exosome complex. *PLoS Pathog.* 11, e1004780 (2015).

21. Kitamura, K. *et al.* Uracil DNA glycosylase counteracts APOBEC3G-induced hypermutation of hepatitis B viral genomes: excision repair of covalently closed circular DNA. *PLoS Pathog.* 9, e1003361 (2013).

37. Ahasan, M, M. *et al*. APOBEC3A and 3C decrease human papillomavirus 16 pseudovirion infectivity. Biochem. Biophys. Res. Commun. 457, 295–299 (2015).
